# Supplementary material for: Complete protection by a single-dose skin patch–delivered SARS-CoV-2 spike vaccine
Source: Sci Adv. 2021 Oct 29;7(44):eabj8065. doi: 10.1126/sciadv.abj8065 (PMC8555896; doi:10.1126/sciadv.abj8065)
Supplement: Supplementary file 1 — Figs. S1 to S6 [file sciadv.abj8065_sm.pdf]

Science Advances
AAAS

Supplementary Materials for

Complete protection by a single-dose skin patch–delivered SARS-CoV-2 spike vaccine

Christopher L. D. McMillan, Jovin J. Y. Choo, Adi Idris, Aroon Supramaniam, Naphak Modhiran, Alberto A. Amarilla, Ariel Isaacs, Stacey T. M. Cheung, Benjamin Liang, Helle Bielefeldt-Ohmann, Armira Azuar, Dhruba Acharya, Gabrielle Kelly, Germain J. P. Fernando, Michael J. Landsberg, Alexander A. Khromykh, Daniel Watterson, Paul R. Young, Nigel A. J. McMillan, David A. Muller*

*Corresponding author. Email: d.muller4@uq.edu.au

Published 29 October 2021, *Sci. Adv.* **7**, eabj8065 (2021)
DOI: 10.1126/sciadv.abj8065

This PDF file includes:

Figs. S1 to S6

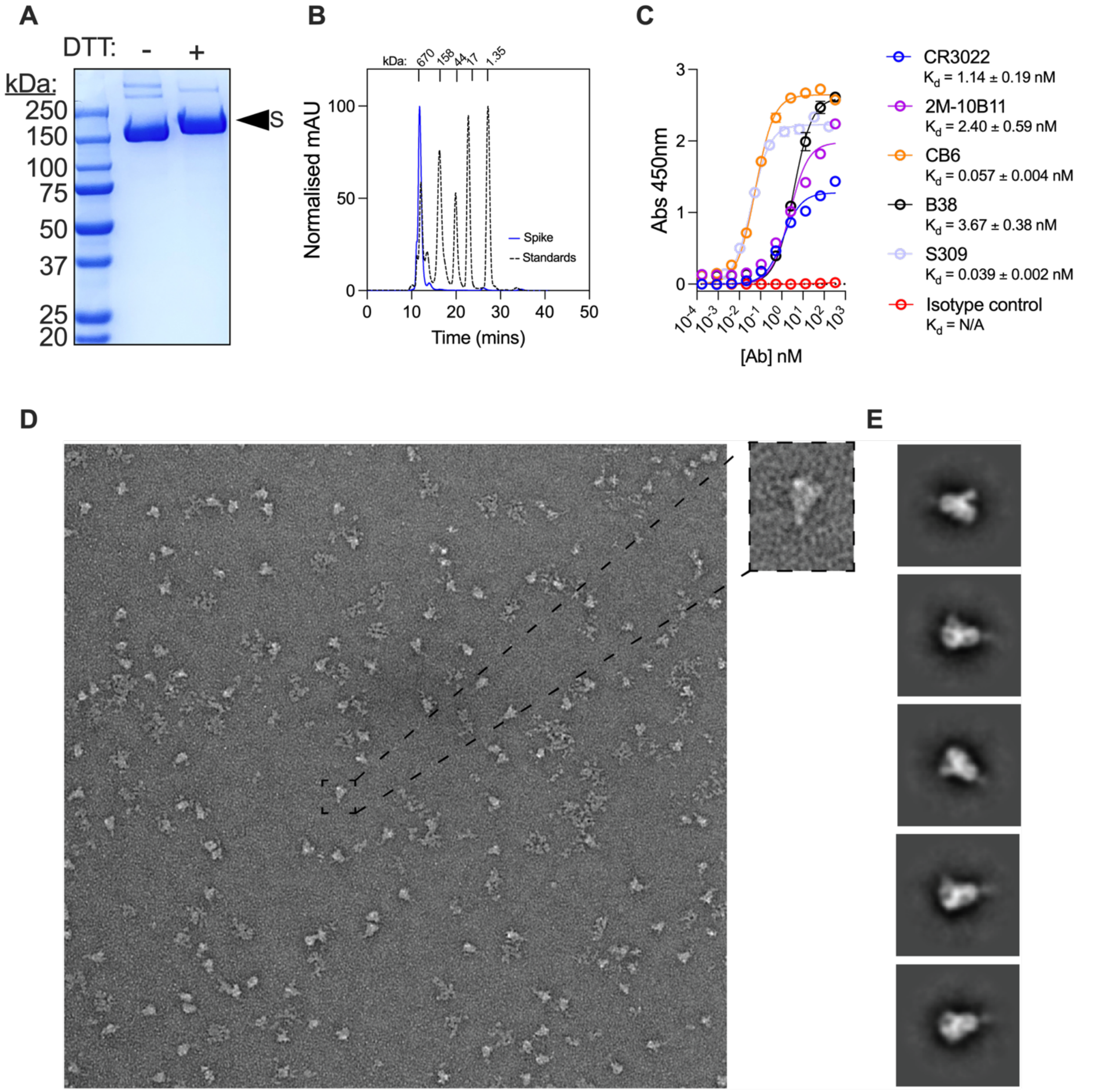

Fig. S1. ***In vitro*** **characterisation of SARS-CoV-2 spike.** **(A)** Coomassie-stained SDS-PAGE of purified SARS-CoV-2 S HexaPro with or without DTT. **(B)** Analytical size-exclusion chromatography of purified HexaPro spike on a Superdex 200 Increase 10/300 GL column, including size markers. **(C)** ELISA of purified HexaPro spike with a panel of SARS-CoV-2 spike-specific monoclonal antibodies. Data represents mean of n=2 technical replicates and error bars represent SD. **(D)** Negative-stain TEM of purified HexaPro spike with representative 2D class averages shown in **(E)** with a box size of 500Å.

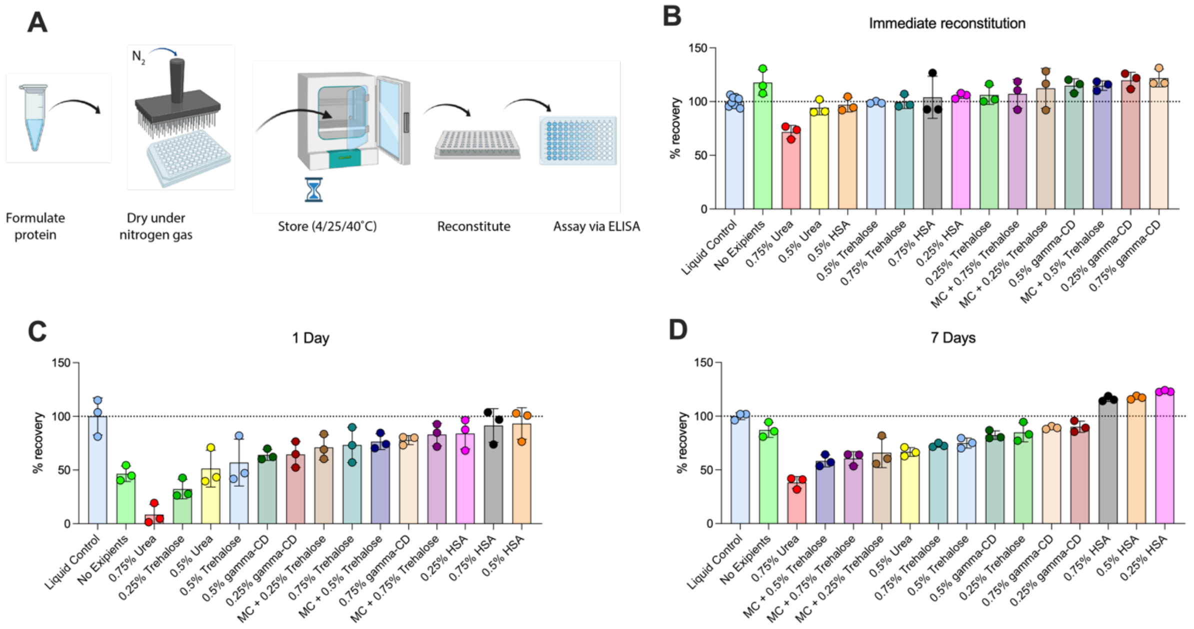

Fig. S2. Stability of dried spike. (A) Schematic of the process of excipient screening. SARS-CoV-2 spike was dried in various excipients and reconstituted **(B)** immediately or stored at 4 ˚C for **(C)** 1 or **(D)** 7 days prior to reconstitution. Recovered protein was analysed via ELISA with the conformation dependent S-specific mAb S309. Percent recovery is relative to a liquid control prepared fresh on the day of each assay.

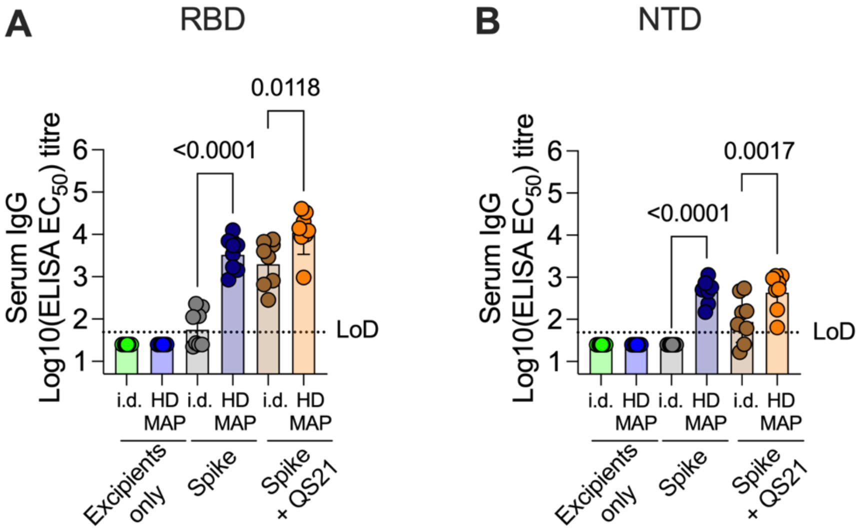

Fig. S3. Domain-specific IgG titers. Serum from mice immunized with SARS-CoV-2 spike was assessed by ELISA against **(A)** the receptor binding domain (RBD) or **(B)** N-terminal domain (NTD) of the spike protein. Data representative of geometric mean with error bars representing geometric SD. *P* values indicate results of one-way ANOVA with Tukey's multiple comparison *post-hoc* test.

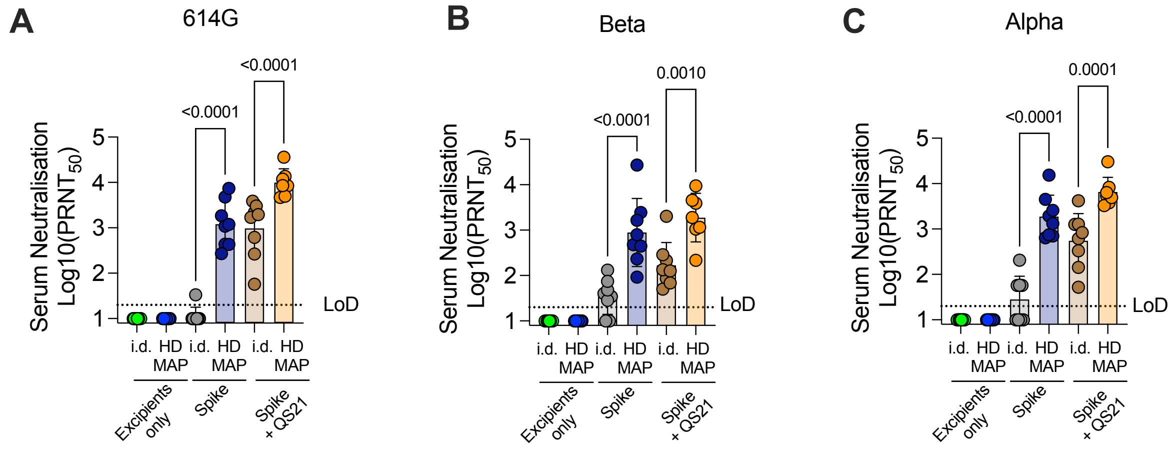

Fig. S4. Neutralization of SARS-CoV-2 variants. Serum from mice immunized with SARS-CoV-2 spike via intradermal (i.d.) delivery or HD-MAP delivery, with or without QS21 as an adjuvant, was assessed for neutralization against SARS-CoV-2 variants **(A)** containing the 614G mutation in the spike protein, **(B)** a Beta variant (lineage B.1.351) and **(C)** an Alpha variant (lineage B.1.1.7). Data representative of geometric mean with error bars representing geometric SD. *P* values indicate results of one-way ANOVA with Tukey's multiple comparison *post-hoc* test.

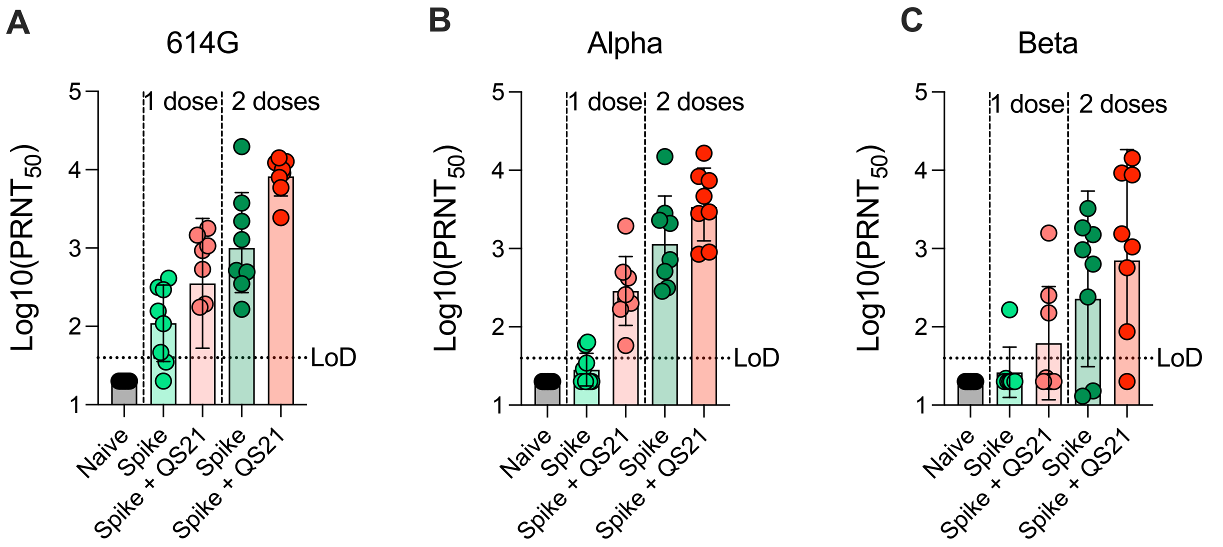

Fig. S5. SARS-CoV-2 variant neutralization by K18-hACE2 mouse serum. Serum from K18-hACE2 mice immunized with 1 or 2 doses of HD-MAP delivered spike was analysed for neutralization by PRNT against SARS-CoV-2 variants **(A)** 614G, **(B)** Alpha (from the B.1.1.7 lineage) and (**C**) Beta (from the B.1.351 lineage). Data represents geometric mean and error bars represent geometric SD.

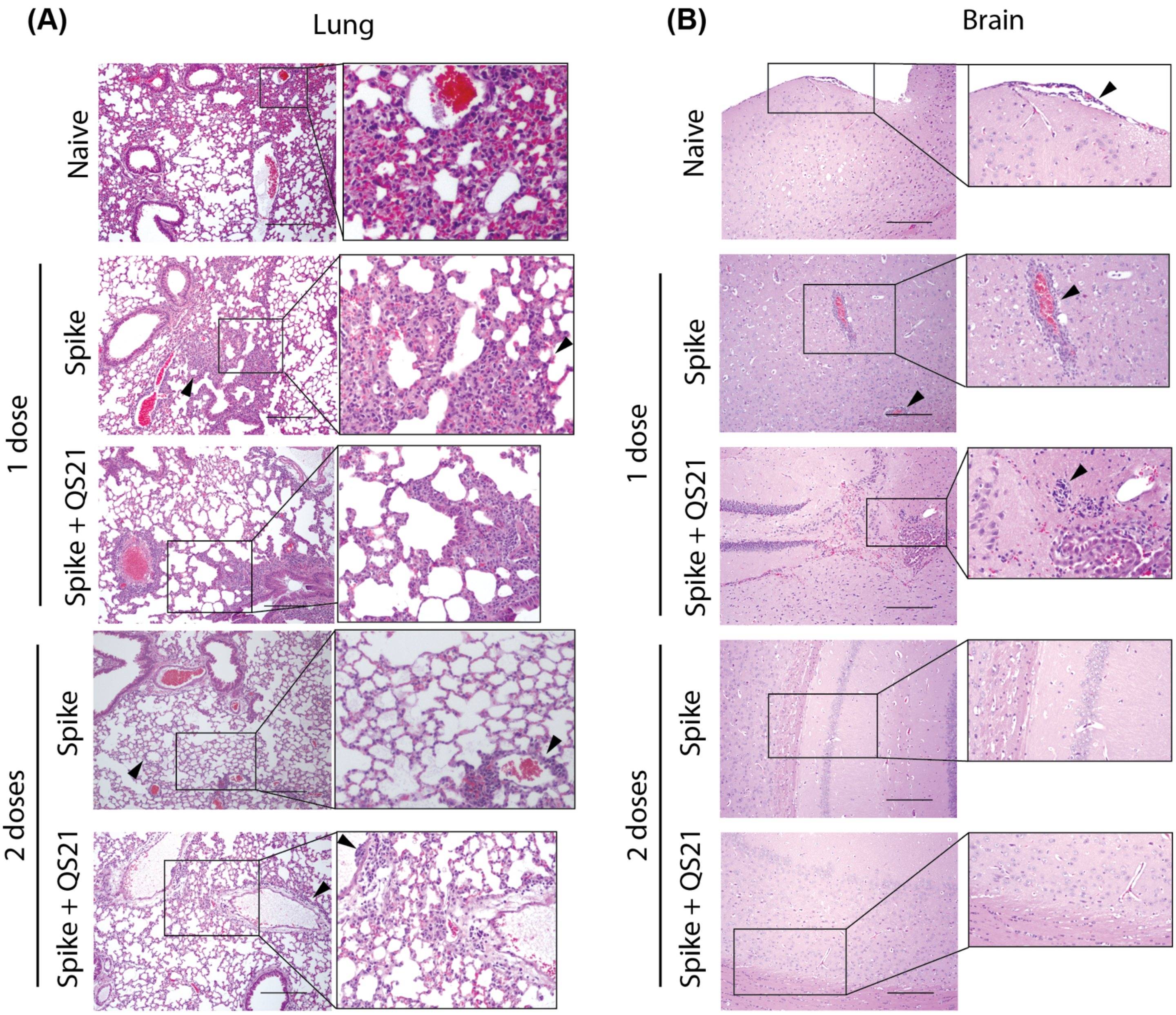

Fig. S6. Histopathology of lungs and brains from K18-hACE2 challenged with SARS-CoV-2. (A) Lungs and **(B)** brain tissue from naïve or vaccinated K18-hACE2 mice challenged with SARS-CoV-2 were collected on day 6 post-infection for hematoxylin and eosin staining. Representative images are shown. Arrowheads indicate leukocyte infiltration (lungs) or perivascular cuffing (brain).
